# Successful Bronchial Occlusion With an Endobronchial Watanabe Spigot Using a Single‐Use Bronchoscope for Bronchobiliary Fistula: A Case Report

**DOI:** 10.1002/rcr2.70376

**Published:** 2025-10-28

**Authors:** Nanako Akiyama, Hiroki Wakabayashi, Ryogo Ohashi, Kensuke Namba, Misa Iwayanagi, Hiromasa Sakurai, Yusuke Irie, Daiki Sakai, Kenta Takashima, Yu Murakami, Masaru Tsuchiya, Kaichi Kaneko, Kazutoshi Isobe, Yasuo Matsuzawa

**Affiliations:** ^1^ Division of Respiratory Medicine, Department of Internal Medicine Toho University Sakura Medical Center Sakura Chiba Japan; ^2^ Department of Surgery Toho University Sakura Medical Center Sakura Chiba Japan

**Keywords:** bronchial occlusion, bronchobiliary fistula, case report, single‐use bronchoscope

## Abstract

Bronchobiliary fistula (BBF) is a rare condition characterised by a pathological communication between the biliary tract and bronchial tree secondary to various diseases. Treatment includes bronchial occlusion using reusable bronchoscopes, although the use of single‐use bronchoscopes (SUBs) for this purpose has not been reported. This case report describes a 62‐year‐old male who developed a BBF after anterior hepatic segmentectomy for hepatocellular carcinoma. The patient was admitted to the intensive care unit for severe pneumonia and respiratory failure. Bedside bronchoscopy using SUBs revealed bile leakage from the right B7 bronchus, leading to the diagnosis of BBF. Due to his critical condition and transport difficulties, bronchial occlusion was successfully performed at bedside using an SUB. The patient was extubated on day 12 and discharged on day 25 without recurrence. This case highlights the utility of SUBs for diagnostic and therapeutic intervention in critically ill patients.

## Graphical abstract

A rare case of bronchobiliary fistula after liver resection was successfully diagnosed and treated with bronchial occlusion at bedside using a single‐use bronchoscope. The use of single‐use bronchoscopes may be viable in critically ill patients, especially when the use of a reusable bronchoscope is not feasible.

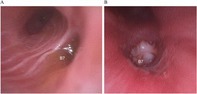

## 1 Introduction

Bronchobiliary fistula (BBF) is a rare disease characterised by abnormal communication between biliary tract and bronchial tree, usually secondary to carcinoma, biliary stenosis, hepatic hydatidosis, cholangiolithiasis, or trauma [1, 2]. Bile leakage into the airways can cause biliptysis, pneumonia, or respiratory failure [2]. Treatment strategies include conservative management (i.e., biliary drainage and infection control), endoscopic retrograde cholangiopancreatography with stenting, percutaneous transhepatic biliary drainage, and surgical repair. Bronchial occlusion with an Endobronchial Watanabe Spigot (EWS) has recently emerged as a new treatment option [1].

Single‐use bronchoscopes (SUBs) are used in settings where access to standard equipment may be limited, such as in the intensive care unit (ICU) or operating room [3]. Due to advancements in image quality and availability, SUBs have been used with high physician satisfaction and are increasingly utilised for diagnostic and therapeutic procedures [3], but limited evidence supports its use in bronchial occlusion. This case report describes a case of BBF after liver resection, which was successfully diagnosed and treated with EWS using a SUB at bedside.

## 2 Case Report

**FIGURE 1:**
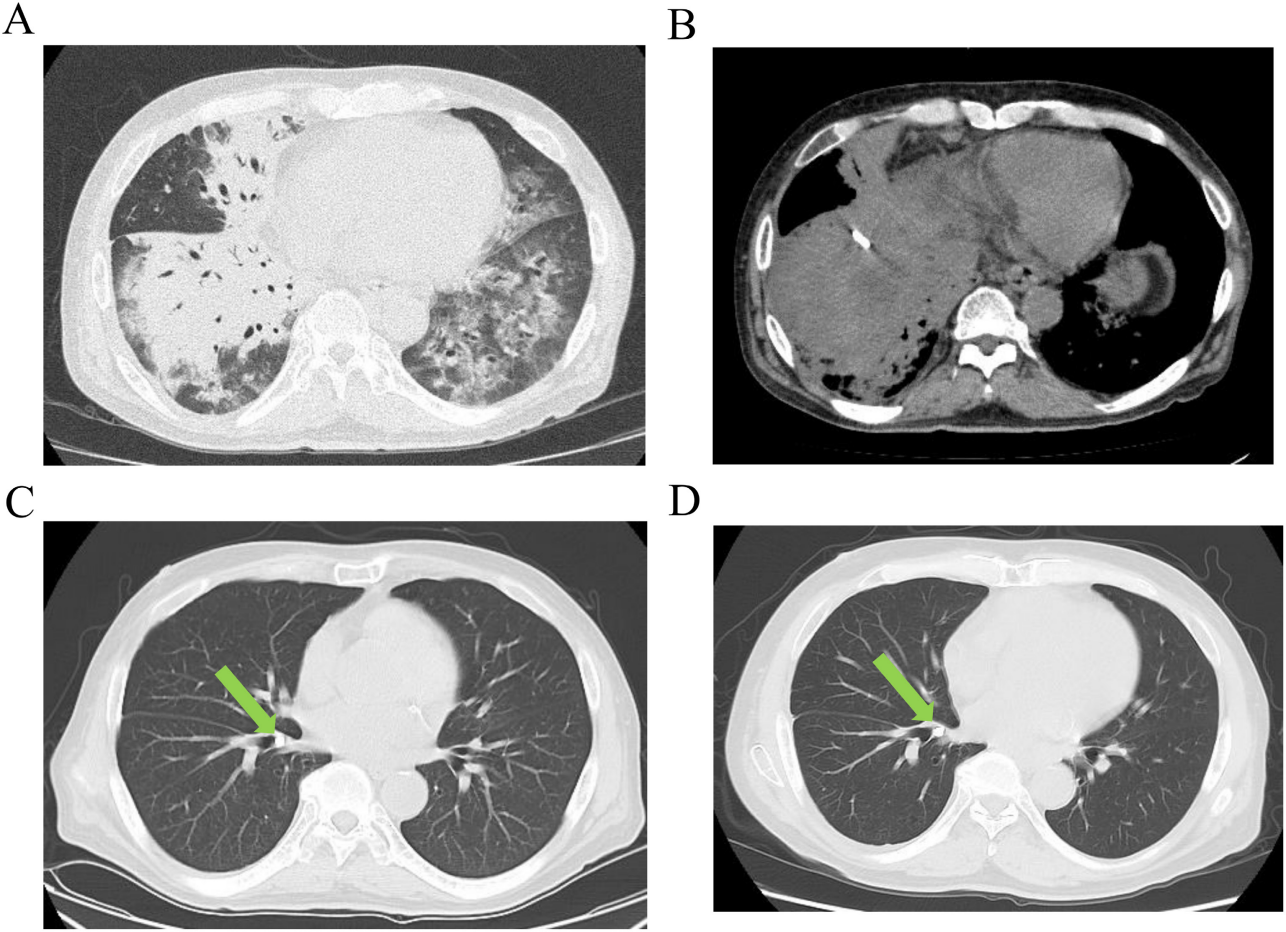
(A) Chest computed tomography (CT) upon admission showing bilateral pulmonary infiltrates, predominantly in the right lung. (B) Abdominal CT upon admission demonstrating an indwelling abdominal drain located immediately below the diaphragm. (C) Chest CT upon discharge revealing marked resolution of the pulmonary infiltrates. The Endobronchial Watanabe Spigot (EWS) is clearly visualised within the right B7 bronchus (arrow). (D) Chest CT at 6‐month follow‐up showing no recurrence of pneumonia. The EWS remains securely in place in the right B7 bronchus (arrow).

A 62‐year‐old male underwent anterior hepatic segmentectomy for hepatocellular carcinoma. Due to postoperative biliary leakage, the patient was managed with prolonged abdominal drainage. He presented at our outpatient clinic complaining of fever, dyspnea, and brownish sputum. The patient had a height of 168.7 cm, weight of 77.3 kg, and body mass index of 27.2 kg/m^2^. The patient was febrile (38.7°C), tachycardic (114 bpm), and hypoxemic (80% oxygen saturation on room air), with a blood pressure of 126/61 mmHg. Physical examination revealed coarse crackles in both the anterior lung fields. Laboratory tests revealed elevations in serum C‐reactive protein (14.78 mg/dL), white blood cell count (15,190/μL), and total bilirubin (1.9 mg/dL). Chest computed tomography (CT) demonstrated an indwelling abdominal drain positioned immediately below the diaphragm with surrounding fluid collection, as well as bilateral pulmonary infiltrates, predominantly in the right lung (Figure 1A,B). Severe bilateral pneumonia was diagnosed.

**FIGURE 2:**
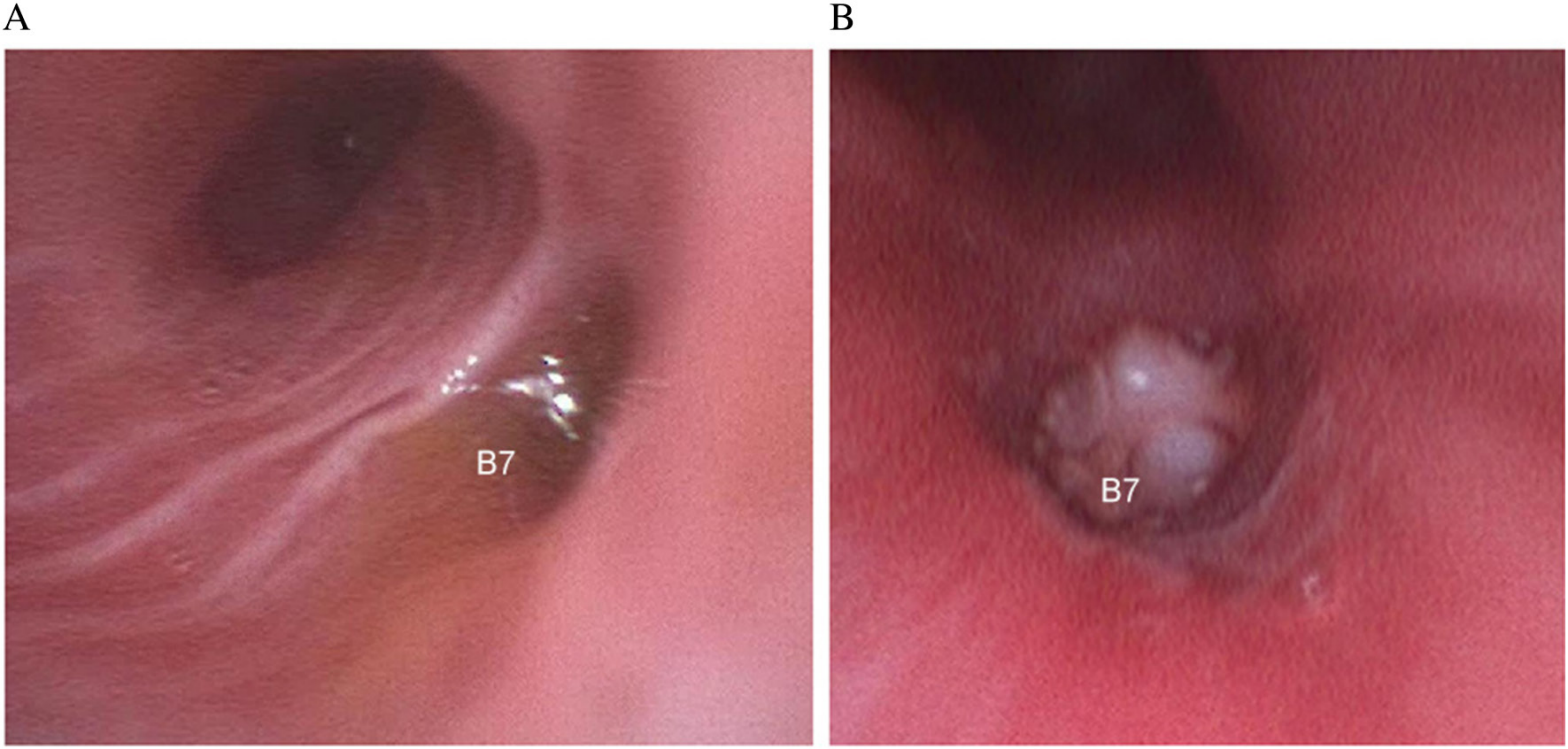
(A) Bronchoscopic image obtained on day 1 revealing active bile leakage from the right B7 bronchus. (B) Bronchoscopic view after successful placement of a manually trimmed medium‐sized EWS at the orifice of the right B7 bronchus.

The patient was emergently intubated and treated with sulbactam/ampicillin (9.0 g/day) and methylprednisolone sodium succinate (125 mg/day) and was admitted to the ICU. Persistent yellowish‐brown sputum was observed in the endotracheal tube. Bedside bronchoscopy performed on day 1 using a SUB (Ambu aScope 4 Broncho Large, 5.8/2.8; Ambu A/S, Ballerup, Denmark) revealed bile leakage from the right B7 bronchus (Figure 2A). To localise the suspected fistula, indigo carmine was injected through the abdominal drain. Bronchoscopic observation revealed the presence of indigo‐coloured discharge from the same bronchus, confirming the diagnosis of BBF. The PaO_2_/FiO_2_ ratio improved from 86.9 on day 1 to 113 on day 6; however, as no further improvement was observed, the patient could not be extubated. Sputum and bronchial washing fluid cultures revealed no pathogenic organisms, and cytologic examination of the bronchial washings showed no malignant cells. Therefore, the respiratory failure was primarily attributed to chemical pneumonitis caused by ongoing bile leakage from the BBF.

On day 7, bronchial occlusion therapy with an EWS was performed at bedside using an SUB. The medium‐sized EWS was manually cut and shaped to match the diameter of the bronchial orifice; this was successfully placed at the entrance of the right B7 bronchus (Figure 2B). Afterward, the patient was successfully extubated on day 12 and transferred out of the ICU. He was discharged home on day 25 without recurrence of symptoms. Follow‐up chest CT demonstrated a correctly placed EWS, resolution of the pulmonary infiltrates, and no evidence of respiratory deterioration (Figure 1C,D).

## 3 Discussion

The patient in this case developed a BBF after hepatic resection, which was successfully diagnosed and treated using SUBs. BBF is a rare complication of hepatic resection and is associated with high morbidity, although its exact incidence remains unclear. A systematic review of 68 cases reported that BBF commonly presents with biliptysis and is typically diagnosed via bronchoscopy or imaging [2]. In this case, SUB enabled prompt bedside identification of the responsible bronchus, which was achieved by injecting indigo carmine through the abdominal drain. This is the first report of successful EWS placement using a SUB.

SUBs have become increasingly suitable for therapeutic interventions, owing to their improved availability, manoeuvrability, channel diameter, and image quality [3]. SUBs have received favourable evaluations for performance and sampling, even in simulated bronchoalveolar lavage procedures using low‐fidelity lung models. This case demonstrates the practicality and utility of SUBs for procedures requiring fine manipulation, such as bronchial occlusion.

In this case, bronchial occlusion was performed using an EWS, which is one of the treatment options for BBF in patients who are poor surgical candidates. The EWS is a silicone bronchial plug that is inserted into the affected airway by grasping it with forceps passed through the bronchoscope's working channel. The plug is cylindroconical in shape, with its distal short axis designed to engage the bronchial lumen. Six small hemispherical surface projections on its body provide frictional fixation within the airway. Zhao et al. reported a case series of four patients who developed BBF after treatment for hepatocellular carcinoma. Treatment with bronchial occlusion resulted in clinical improvement in three patients (75%) [1], although 2 to 6 sessions were needed to achieve resolution. In the present case, only a single session of bronchial occlusion was needed to successfully treat the patient without recurrence of pneumonia. This favourable outcome can be attributed to three key factors. First, the responsible bronchus was clearly identified using indigo carmine. Second, the fistula was limited to the right B7 bronchus, facilitating a focused intervention. Third, the EWS was carefully trimmed to fit the bronchial lumen, effectively sealing the leakage site. Additionally, compared to conventional reusable bronchoscopes, the SUBs used in our case were more flexible and easier to manipulate.

Bronchial occlusion carries risks such as atelectasis, infection, hypoxemia, and post‐procedural device migration or expectoration. In this case, we mitigated these risks, particularly the risk of dislodgement, by trimming the EWS to achieve a patient‐specific press‐fit. The nominal EWS dimensions are as follows: small, 4 × 5 × 12 mm; medium, 5 × 6 × 14 mm; and large, 6 × 7 × 16 mm (short axis × long axis × length). In our patient, the medium spigot was initially too large to seat securely, whereas the small spigot was undersized and prone to dislodgement. We therefore trimmed approximately 2 mm from the distal short axis of the medium‐size spigot, which enabled a snug, stable fit within segment B7.

Furthermore, by localising the fistula exclusively to B7 and avoiding unnecessary occlusion of adjacent bronchi, we likely minimised the risks of atelectasis, secondary infection, and hypoxemia. Although BBF is rare and evidence regarding optimal treatment strategies is limited, our case demonstrates the feasibility and practicality of bedside bronchial occlusion using an SUB and EWS in critically ill patients.

## Author Contributions

**Nanako Akiyama:** writing – original draft, data curation. **Hiroki Wakabayashi:** writing – original draft, project administration. **Kensuke Namba:** writing – review and editing. **Ryogo Ohashi:** writing – review and editing. **Misa Iwayanagi:** writing – review and editing. **Hiromasa Sakurai:** writing – review and editing. **Daiki Sakai:** writing – review and editing. **Yusuke Irie:** writing – review and editing. **Kenta Takashima:** writing – review and editing. **Yu Murakami:** writing – review and editing. **Masaru Tsuchiya:** writing – review and editing. **Kaichi Kaneko:** writing – review and editing. **Kazutoshi Isobe:** project administration and conceptualization. **Yasuo Matsuzawa:** supervision.

## Consent

Written informed consent was obtained for the publication of this manuscript and accompanying images. The form used to obtain consent from the patient complies with the journal requirements.

## Conflicts of Interest

The authors declare no conflicts of interest.

## Data Availability

Data sharing not applicable to this article as no datasets were generated or analysed during the current study.
